# Antimicrobial polycarbonates for biomedical applications

**DOI:** 10.1186/1878-5085-5-S1-A133

**Published:** 2014-02-11

**Authors:** Sergiy P Rogalsky, Olena V Moshynets, Lyudmila G Lyoshina, Oksana P Tarasyuk

**Affiliations:** 1 Institute of Bioorganic Chemistry and Petrochemistry of NAS of Ukraine, Kyiv, Ukraine; 2 Institute of Molecular Biology and Genetics of NAS of Ukraine, Kyiv, Ukraine

## Background

Polycarbonate (PC) is one of the most widely used engineering polymers due to its unusual combination of optical clarity, heat resistance, high impact strength and dimensional stability over wide thermal range [1]. Low water absorption, ease of sterilization and biocompatibility of PC have led to its use in a wide range of medical equipment, including critical medical devices [2]. The prevention of biofilm formation on the internal medical devices is of great importance since it can initiate a degradation process of the material, as well as cause infections and health related problems [3]. In order to obtain antimicrobial properties, polymers are usually compounded with organic or inorganic biocides [4,5]. However, the use of organic biocides as additives for PC is significantly limited due to their insufficient thermal stability in the temperature range used for PC processing (300 – 320 °C).

## The aim

of research was to develop novel low toxic biocides for PC having sufficient thermal stability for the joint melt processing with PC resin.

## Materials and methods

Two kinds of potential antimicrobial additives for PC have been synthesized: imidazolium and guanidinium ionenes, as well as imidazolium based ionic liquids. PC films containing from 1 to 10 wt% of biocides were prepared by solvent casting or by compression molding methods. The thermal stability of novel biocides as well as modified PC was investigated by using thermogravimetric analysis (TGA). Antimicrobial testing of PC composites were performed using a model bacteria *Pseudomonas fluorescens* SBW25 with a help of classical microbiological methods for bacterial suspension as well as a live-dead assay for biofilms [6].

## Results

According to TGA data, the thermal stability of PC samples containing biocidal additives was found to be in the range of 350-425 ^o^C that is quite sufficient for their melt processing. The results of microbiological investigations showed pronounced antimicrobial efficacy of PC films containing 3-4 wt% of ionic liquids or 5-8 wt% of ionene polymers.

## Conclusions

Imidazolium ionic liquids and ionene polymers are promising antimicrobial additives for PC resins since they combine high thermal stability, low toxicity and broad spectrum of antimicrobial activity.

## Outlook and expert recommendations

According to the obtained results we recommend to expand the application of polimers to design polycarbonate based medical devices for prevention of postoperative infectious complications in orthopedics after performing relevant biosafety and preclinical tests.
